# Recognizing a Heart Attack: Patients’ Knowledge of Cardiovascular Risk Factors and Its Relation to Prehospital Decision Delay in Acute Coronary Syndrome

**DOI:** 10.3389/fpsyg.2020.02056

**Published:** 2020-08-25

**Authors:** Dunia Garrido, Dafina Petrova, Andrés Catena, José Antonio Ramírez-Hernández, Rocio Garcia-Retamero

**Affiliations:** ^1^Faculty of Health Sciences, Universidad de Castilla-La Mancha, Toledo, Spain; ^2^Mind, Brain, and Behavior Research Center (CIMCYC), University of Granada, Granada, Spain; ^3^Escuela Andaluza de Salud Pública, Granada, Spain; ^4^Instituto de Investigación Biosanitaria ibs.Granada, Granada, Spain; ^5^CIBER of Epidemiology and Public Health (CIBERESP), Madrid, Spain; ^6^Cardiology Department, Virgen de las Nieves University Hospital, Granada, Spain; ^7^Harding Center for Risk Literacy, Max Planck Institute for Human Development, Berlin, Germany

**Keywords:** acute coronary syndrome, patient decision making, prehospital delay, knowledge, decision delay, heart attack, cardiovascular risk

## Abstract

In acute coronary syndromes (ACSs), longer decision delay – the time patients wait before seeking medical attention after symptoms have started – increases the risk of complications and death. However, many patients wait much longer than recommended and research is needed investigating how patient decision delay can be reduced. In a cross-sectional study of 120 ACS survivors, we investigated the relationship between knowledge of cardiovascular risk factors and decision delay. Several days after the onset of a cardiac event, patients completed a questionnaire measuring demographics, decision delay, objective knowledge of cardiovascular risks factors and of ACS symptoms, and subjective perceptions of symptoms during the cardiac episode. Relevant clinical data were extracted from patients’ medical records. In a multiple linear regression analysis, controlling for demographic and clinical factors, objective knowledge of cardiovascular risk factors and ACS symptoms, and subjective attributions of symptoms to a cardiac cause were related to shorter decision delays. Among patients with relatively high knowledge of risk factors, only 5% waited more than 1 h to seek help, compared to 22% among patients with relatively low knowledge. These results suggest that knowledge of the factors that increase the risk of developing cardiovascular disease could play a role in patient decision making during an acute cardiac event. We discuss methodological issues and potential underlying mechanisms related to decision heuristics and biases, which can inform future research.

## Introduction

Cardiovascular disease is the most common cause of death worldwide, responsible for 25% of deaths in Europe and causing more premature deaths than cancer (Heron, 2016; World Health Organization, 2016; World Health Statistics, 2018). The majority of deaths from cardiovascular disease are due to coronary heart diseases including acute coronary syndromes (ACSs) – responsible for 43% of deaths due to cardiovascular disease (Turpie, 2006; American Heart Association, 2016; World Health Statistics, 2018). ACSs usually manifest with chest pain or discomfort, pain in one or both arms, pain in the jaw, neck, back, or stomach, and shortness of breath, among others.

Rapid action is crucial in the management of ACS, because a longer prehospital delay – referring to the time from symptom onset to receiving treatment – has been linked to worse clinical outcomes and increased mortality (Rollando et al., 2012; Guerchicoff et al., 2014). However, results of previous interventions aiming to reduce patients’ prehospital delay were mixed and it is not clear what components of these interventions increased their success (Mooney et al., 2012; Farquharson et al., 2019). Further research is needed to shed light on the factors that could reduce prehospital delays and thus improve patient outcomes.

Previous research has investigated the effect of socio-demographic, clinical, and situational factors on prehospital delay. For instance, older adults, females, patients with relatively low socioeconomic backgrounds and those with chronic diseases have longer prehospital delays (Moser et al., 2006; Khraim and Carey, 2009; Wechkunanukul et al., 2017). Similarly, patients who live alone or are alone at symptom onset, patients who do not call an ambulance but consult with a physician, and those who suffer the cardiac episode during daytime also have longer prehospital delays (Moser et al., 2006; Wechkunanukul et al., 2017).

A large body of research has also investigated cognitive and emotional factors related to prehospital delays. To illustrate, a recent systematic review of 57 studies conducted in 23 countries concluded that social concerns such as embarrassment in asking others for help or worry about troubling others were not systematically related to prehospital delays (Arrebola-Moreno et al., 2020a). In contrast, patients who attributed symptoms to a cardiac cause, perceived symptoms as serious, and felt anxiety in response to symptoms report shorter prehospital delays (Arrebola-Moreno et al., 2020a). Overall this literature indicates that symptom attribution to cardiac as opposed to other causes such as muscular, respiratory or digestion problems, is fundamental to speed up help-seeking.

**FIGURE 1:**
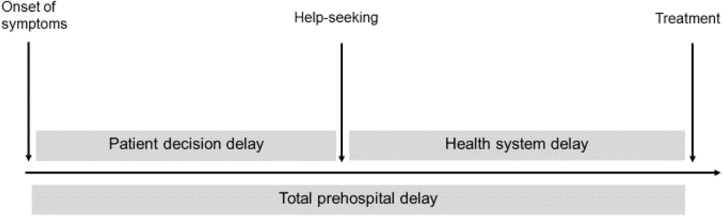
Phases of prehospital delay.

Several studies showed that patient *decision* delay – the time elapsed between symptom onset and the moment patients decide to seek medical attention – is one of the major contributors to prehospital delays (see Figure 1; Ottesen et al., 2004; Moser et al., 2006; Mackay et al., 2014; Wechkunanukul et al., 2017). Thus, a potentially effective strategy for reducing prehospital delays would be to improve patient decision making. However, most previous studies measured total prehospital delay without differentiating the patient decision delay component (Mackay et al., 2014). In fact, reviews show that only between 18 and 33% of studies report patient delays in decision making (Mackay et al., 2014; Arrebola-Moreno et al., 2020a). This is an important shortcoming because factors such as patient knowledge or perceptions are unlikely to influence health system delays; thus, considering total prehospital delay instead of only patient decision delay to study the influence of patient-related factors introduces avoidable error variance. In the current research, we investigated patient decision delay and we focused on its relationship with patients’ knowledge and perceptions.

To be able to assign the experienced symptoms to a heart problem such as ACS, patients would need to know what the typical symptoms of ACS are. Such symptom knowledge is usually assessed with objective measures (i.e., patients’ correct recognition of the symptoms in a test-like questionnaire) or subjective measures (patients’ self-reported knowledge of the symptoms before the cardiac event). However, both types of measures have shown mixed results in relation to prehospital delays (Arrebola-Moreno et al., 2020a).

It is possible that symptom knowledge is not sufficient to speed up decision making if individuals do not know that they are at risk of suffering an ACS. There are multiple risk factors that make it more likely to suffer coronary heart disease including older age, smoking, diabetes, hypertension, and obesity (American Heart Association, 2016). However, research shows that people are not generally aware of these risk factors (Erhardt and Hobbs, 2002; Jensen and Moser, 2008; Wartak et al., 2011), and those with relatively low knowledge may underestimate the probability of experiencing a cardiac event (Lefler and Bondy, 2004; Darawad et al., 2016).

In the current research, we investigated for the first time whether knowledge about cardiovascular risk factors is related to decision delay in patients experiencing an ACS. The focus on decision delay rather than total prehospital delay would be particularly relevant for the current research. The rationale is that the latter is not only influenced by patients’ decision making but also by other factors that are out of patients’ control (e.g., health system delays). To account for other factors that could influence patient decision making (Nguyen et al., 2010; Arrebola-Moreno et al., 2020a), we also investigated the effect of patients’ objective knowledge of ACS symptoms, subjective attributions of symptoms to cardiac causes, perceived severity of symptoms, and demographic factors. Our hypothesis was that patients’ knowledge about cardiovascular risk factors would be uniquely related to decision delay after accounting the effect of the other factors.

## Materials and Methods

This was a cross-sectional retrospective study of ACS patients admitted to the Cardiology Department of the University Hospital Virgen de las Nieves (Granada, Spain) who underwent a percutaneous coronary intervention (PCI) as part of the management of ACS between March 2017 and April 2019. The study was completed on average 4.67 days (95% CI 4.24–5.09) after the cardiac event.

All participants signed an informed written consent before participation and the Hospital Ethics Committee approved the study. The inclusion criteria were: (a) having been diagnosed with an ACS, (b) being younger than 75, and (c) being fluent in Spanish. The exclusion criteria were having an inflammatory disease or a neurological problem that prevented participation in the study. Patients were selected based on these criteria by a qualified cardiologist who extracted information about the final diagnosis. To minimize the exclusion of participants due to fatigue, illiteracy, or other reading difficulties the researcher offered help to all patients and gave detailed instructions.

Participants completed a survey that started with assessment of standard data for studies in ACS patients (demographics, family history of cardiovascular disease, anthropometric data, and healthy habits). Participants then completed the measures described below, including knowledge of ACS symptoms, knowledge of CV risk factors, prehospital delay, and part of the modified Response to Symptoms Questionnaire (based on Burnett et al., 1995; Dracup and Moser, 1997)^1^.

*A priori* analysis with G^∗^power^2^ assuming alpha = 0.05, power = 0.80, and a total of 10 predictors indicated that to detect an effect size of *R*^2^ = 0.06 for one tested predictor 126 participants would be required (see OSF: 10.17605/OSF.IO/CEHN7). The choice of effect size was based on the average documented effect size of diverse psychological factors on prehospital decision delay in a previous study in this population (*R*^2^ between 0.05 and 0.07) (Arrebola-Moreno et al., 2020b). Because we expected some participant attrition (e.g., due to missing clinical records, incomplete questionnaires or final diagnosis determined not to be ACS), we decided to invite a minimum of 150 patients (+20% of the required sample size).

### Measures

#### Clinical Information

The following measures were obtained from patients’ medical records: (a) number of days elapsed from cardiac event to completion of the questionnaire, (b) cardiovascular disease history – e.g., any previous myocardial infarction or ischemic disease, (c) smoking – i.e., non-smoker or smoker, (d) history of diabetes, (e) history of hypertension, (f) body mass index – i.e., weight (kg)/height (m)^2^, (g) type of myocardial infarction – i.e., ST-segment elevation myocardial infarction (STEMI) or a non-STEMI, (h) obstructed arteries – i.e., number of obstructed vessels, (i) ejection fraction (EF) – i.e., the amount of blood that is pumped out of the ventricles, considering an EF of <35%, 35–45%, 45–55%, and >55% as very reduced, moderately reduced, slightly reduced, and normal respectively, and (j) revascularization – i.e, complete or incomplete revascularization.

#### Decision Delay

It was calculated as the time difference, in minutes, between symptom onset and the patients’ decision to seek medical attention. Patients were asked to determine (1) at what time symptoms started and (2) at what time they decided to seek medical attention (e.g., when they decided to go to the hospital or call an ambulance), and we computed the difference between the two time points. This measure was validated in a previous study against patients’ troponin levels on arrival at the hospital (see Petrova et al., 2017; Arrebola-Moreno et al., 2020b). Troponin is a protein that is released when the heart muscle has been damaged and is currently the gold standard for ACS diagnosis and management (ECS guidelines; Roffi et al., 2016). It has a known progression curve that make it a useful additional measure of the time elapsed from ASC onset.

#### Knowledge of Cardiovascular Risk Factors

This was assessed with a questionnaire measuring participants’ knowledge of the effect of 52 factors on the risk of developing cardiovascular disease designed for this research. There were four types of factors: modifiable factors (24 items, e.g., smoking cigarettes and eating fresh vegetables), uncontrollable factors (7 items, e.g., age – e.g., older than 65), psychosocial factors (13 items, e.g., having social support), and fictitious causes/filler items (8 items, e.g., being bitten by a mosquito)^3^. The selection of factors was based on guidelines for the prevention of cardiovascular disease and further scientific literature on risk and protective factors for cardiovascular disease (Winkleby et al., 1992; Myrtek, 2001; Rosengren et al., 2004; Grande et al., 2012; Rozanski, 2014; Khera et al., 2016; Piepoli et al., 2016; World Heart Federation, 2017).

Participants were asked to indicate for each factor what they thought its effect was on the risk of developing cardiovascular disease using a 5-point scale ranging from “it reduces the risk very much” to “it increases the risk very much” with a neutral point indicating that “it has no effect.” Items were scored as correct if patients correctly identified whether the item was a risk factor (it increases the risk), a protective factor (it reduces the risk), or it has no effect. We calculated the sum of the number of correct answers for each category. The final score was the total number of correct answers excluding the filler items (i.e., modifiable + uncontrollable + psychosocial factors).

#### Knowledge of ACS Symptoms

This was measured using the ACS response index (Riegel et al., 2007) that lists 21 predefined symptoms, including arm pain, weakness/fatigue, sweating, and chest discomfort. Patients were asked to indicate whether they thought it was a symptom of heart attack (yes/no) or they did not know. The final score was calculated as the sum of the number of correctly identified symptoms.

#### Modified Response to Symptoms Questionnaire

Participants answered four multiple-choice questions evaluating (a) what symptoms they experienced, (b) where they were when the symptoms started, (c) whom they were with, (d) what they thought the problem was, and (e) the perceived symptom severity (i.e., how severe they thought the symptoms were at onset, ranging from 1 “not at all severe” to 6 “very severe”) (Burnett et al., 1995; Dracup and Moser, 1997). From the responses to (d), the variable “attribution to a cardiac origin” was created, where responses indicating a heart problem were coded as 1 and the rest (e.g., stomach, muscular, dental problems, fatigue, etc.) were coded as 0.

### Data Analyses

First, we describe our sample using descriptive statistics. The variable decision delay was positively skewed, so median and interquartile ranges were considered and the variable was log-transformed for analysis. Second, to investigate the relationship between prehospital decision delay and knowledge of cardiovascular risk factors, knowledge of ACS symptoms, attribution to a cardiac origin, and perceived symptom severity, we computed bivariate Pearson correlations, followed by multiple linear regression analyses.

## Results

During the study period the participating cardiologist identified 207 patients fulfilling the inclusion criteria of which 156 were invited to participate. From these, 140 agreed to participate and 120 returned completed questionnaires. Thus, final sample size was 120 (69.2% male, age μ = 59.87, *SD* = 8.80, range from 41 to 75). Descriptive statistics for all study variables are presented in Tables 1,2.

**TABLE 1:** Demographic and clinical characteristics of the sample (categorical variables).

	**Number**	**Percentage**
Age > 60 years	58	48
Age > 70 years	13	11
Sex: Male	83	69
**Education**		
Low (no or primary education)	66	55
Medium (secondary education)	12	10
High (tertiary education)	42	35
**Acute coronary syndrome severity**		
STEMI	53	41
Ejection fraction		
Very reduced	11	9
Moderately reduced	19	16
Slightly reduced	22	18
Normal	62	52
Complete revascularization	72	60
**Risk lifestyle/classical factors**		
Overweight (BMI ≥ 25)	102	85
Obesity (BMI ≥ 30)	49	41
Smoker	54	45
Cardiovascular disease history	22	18
Diabetes	31	26
Hypertension	63	53
**Modified response to symptoms questionnaire**		
Where were you when symptoms started?		
Home	81	68
Work	7	6
Car	7	6
Public place	15	13
Other	10	8
**Whom were you with when symptoms started?**		
Alone	30	25
Partner	53	44
Relative(s)	19	16
Friend(s)	7	6
Workmate(s)	7	6
Other	4	3
Attributed symptoms to cardiac origin	40	33

**STEMI, ST-segment elevation myocardial infarction; BMI, body mass index.**

**TABLE 2:** Descriptive statistics for the sample (continuous variables).

	**Mean**	***SD***	**Min–max**	**Range**	**Missing (%)**
Age, years SD	59.86	8.80	41.00–75.00	–	1 (0.8)
Obstructed arteries	1.53	0.85	0–3	–	5 (4)
BMI, kg/m^2^	29.33	4.94	20.31–53.53	–	0 (0)
Decision delay*	60.00	133.75	1.00–1440.00	–	4 (3)
**Knowledge of cardiovascular risk factors**					
Modifiable factors	20.37	2.05	13–23	0–24	5 (4)
Uncontrollable factors	5.19	0.95	2–7	0–7	5 (4)
Psychosocial factors	9.54	2.01	0–13	0–13	5 (4)
Total risk factors	39.58	3.76	23–45	0–44	5 (4)
Fictitious causes	3.52	1.29	1–7	0–7	4 (3)
**Knowledge of ACS symptoms**					
ACS symptoms	12.67	4.03	0–20	0–21	0 (0)
**Modified response to symptoms questionnaire**					
Perceived symptom severity	3.67	1.71	0–6	0–6	4 (3)

*Decision delay is presented as median and interquartile range; BMI, body mass index.

### Patient Characteristics

As is typical for ACS patients, participants had characteristics consistent with high cardiovascular risk (see Table 1). The majority of patients were males, over 60 years, overweight, and with a history of hypertension. Forty-five percent were smokers, 26% had diabetes, and 18% had previous history of cardiovascular disease. In fact, considering age and the classical risk factors in Table 1, 98% of patients had at least one relevant cardiovascular risk factor, which put them at high cardiovascular risk; in particular, 8% had one, 28% had two, 19% had three, and 43% four or more risk factors.

### Decision Making During the Cardiac Episode

From the whole sample, 40% (*N* = 48) reported a decision delay less or equal to 30 min; 16.7% (*N* = 20) reported a delay between 30 and 60 min; the remaining 43.3% (*N* = 52) reported a delay longer than 60 min. The majority of patients were at home, alone or with a partner when symptoms started; and only 33% correctly attributed symptoms to a cardiac cause (Table 1).

### Knowledge

Overall knowledge of ACS symptoms was low-to-average, with 53% of patients correctly identifying fewer than 14 out of 21 symptoms (see also Table 2). In contrast, knowledge of cardiovascular risk factors was relatively high, with a median of 41 (out of 44). The percentages of patients giving correct answers to each item from the risk factors questionnaire are presented in Table 3. Participants correctly recognized most of the modifiable risk and protective factors but only a few of the uncontrollable and psychosocial factors (although recognition was still high on average). Among the less recognized factors were HDL cholesterol, ethnicity, and locus of control. Importantly, age (one of the most influential risk factors) and gender were only recognized by 71 and 60% of patients, respectively. The fictitious causes subscale revealed that many patients incorrectly thought that factors that have a role in other diseases (e.g., cancer, infectious diseases) were related to cardiovascular disease.

**TABLE 3:** Knowledge of cardiovascular risk factors questionnaire: item responses.

**Factor**	**Effect**	**Item text**	**Correct answer**	
			***N***	***%***
**Modifiable factors**				
Obesity	R	Suffering obesity	113	94
Tobacco consumption	R	Smoking cigarettes	111	93
A diet high in salt	R	Eating food with lots of salt	108	90
Raised blood glucose	R	Having high blood sugar levels (glucose)	108	90
A diet high in saturated fats	R	Having a diet rich in saturated fats (e.g., butter, cream, pastries, processed meat)	108	90
A diet high in trans fats	R	Eating foods high in trans fats (e.g., hamburgers, cakes, chips)	107	89
Mediterranean diet	P	Following the Mediterranean diet: high consumption of vegetable products, bread and other cereals, with olive oil as the main fat.	107	89
High levels of triglycerides	R	Having high triglyceride levels (lipids, a type of blood fat)	107	89
Hypertension	R	Having hypertension (high blood pressure)	106	88
Consumption of fresh vegetables	P	Eating fresh vegetables	105	88
Low density lipoprotein (LDL) levels	R	Having high levels of low density lipoprotein (LDL) (”bad” cholesterol)	104	87
Alcohol consumption	R	Drinking alcohol excessively	104	87
Overweight	R	Being overweight	103	86
Diabetes	R	Having diabetes or prediabetes	102	85
A diet high in omega-3 fatty acids	P	Having a diet rich in omega-3 fats (e.g., fish, nuts)	101	84
Abdominal fat	R	Having a lot of abdominal fat (around the waist)	101	84
Soft sugary drink consumption	R	Drinking sugary drinks (for instance, coke, fanta…)	99	83
Fresh fruit consumption	P	Eating fresh fruits	99	83
Fish consumption	P	Eating fish	92	77
Sitting for prolonged periods of time	R	Spending many hours a day sitting (e.g., watching TV, driving)	91	76
Physical activity	P	Doing physical exercise (walking, running, dancing…)	87	73
High waist-to-hip ratio	R	Having high waist-to-hip ratio (e.g., a prominent belly)	83	69
Fiber consumption	P	Eating foods high in fiber (e.g., legumes, potatoes)	73	61
High-density lipoprotein (HDL) cholesterol	P	Having high HDL (“good”) cholesterol	23	19
**Uncontrollable factors**				
Personal history of CVD	R	Having had cardiovascular disease previously (e.g., a heart attack or stroke)	110	92
Genetic predisposition	R	Genetic predisposition	108	90
Family history of CVD	R	Having a family history (a direct family member who has had or died from cardiovascular disease before age 55)	108	90
Passive smoking	R	Being exposed to tobacco smoke (e.g., being exposed to tobacco smoke from someone who smokes around you)	100	83
Age	R	Being older (por instance, more than 65 years old)	85	71
Sex	R	Being male	72	60
Ethnicity	R	Being form African or Asian ethnicity	17	14
**Psychosocial factors**				
Type-D personality (negative affectivity)	R	Feeling strong negative emotions frequently	106	88
Stress at work	R	Suffering stress at work	105	88
Stress at home	R	Suffering stress at home	104	87
Major stressful life events	R	Having experienced stressful life events in recent years (for instance, going through unemployment, divorce, or the death of a close family member)	104	87
Depression	R	Suffering depression	104	87
Anxiety	R	Suffering frequent anxiety	104	87
Financial stress	R	Suffering economic stress (for instance, not being able to make ends meet, having loans to return)	100	83
Social isolation	R	Feeling alone or socially isolated	93	78
Type-D personality (social inhibition)	R	Not expressing the negative emotions one feels (keeping quiet or not mentioning them)	89	74
Social support	P	Having high social support (support from the people around you)	78	65
Being poor/Low income	R	Having low income	75	63
Education	P	Having high education (for instance, having gone to university and finished one’s studies)	29	24
Locus of control	P	Thinking that you have the control over events that happen around you	7	6
**Fictitious causes**				
Type-A personality (urgency)	NE	Being impatient (e.g., feeling in a hurry and needing to go/act fast frequently)	79	66
Risky sexual behavior	NE	Having unprotected sex with multiple partners	71	59
Pregnancy	NE	Being pregnant (for women)	61	51
Mosquito bites	NE	Being bitten by a mosquito carrying a virus	60	50
Sun exposure	NE	Sunbathing excessively or using sunbeds	57	48
Radiation exposure	NE	Exposure to X-rays and other sources of radiation	37	31
Type-A personality (competitiveness)	NE	Being competitive in everything you do	32	27
Type-A personality (hostility)	NE	Being a hostile person, one who gets angry easily	13	11

**Effect on cardiovascular risk: R, risk factor; P, protective; NE, no effect.**

### Factors Related to Decision Delay

Bivariate Pearson correlations with the log continuous delay score are presented in Table 4. Shorter decision delay was related to more accurate knowledge of cardiovascular risk factors, more accurate knowledge of ACS symptoms, correct attributions of symptoms to a cardiac cause, and higher perceived severity. Those patients who had more accurate knowledge of cardiovascular risk factors also had more accurate knowledge of ACS symptoms. Finally, higher perceived symptom severity was related to accurate attributions of symptoms to a cardiac cause.

**TABLE 4:** Pearson correlations between decision delay with the other variables of interest.

	**1**	**2**	**3**	**4**	**5**	**6**
1. Decision delay (log)	−					
2. Knowledge of CV risk factors	−0.44*	−				
3. Knowledge of fictitious causes	0.09	−0.12	−			
4. Knowledge of ACS symptoms	−0.34*	0.33*	−0.16	−		
5. Attribution to a cardiac cause (1 = yes; 0 = no)	−0.32*	0.18	0.02	−0.00	−	
6. Perceived severity	−0.26*	0.17	0.07	0.13	0.38*	−

*p < 0.01.

For our main analysis, we conducted a multiple linear regression analysis with decision delay as outcome variable. The rest of the variables (i.e., knowledge of cardiovascular risk factors, knowledge of ACS symptoms, attribution to a cardiac cause, and severity of symptoms) were included as predictors. Demographics (age, gender, and education), disease severity (type of ACS), and the number of days elapsed between the cardiac event and completion of the questionnaire were included as controls in this analysis. Knowledge of fictitious factors was not considered in the analysis as it was not related to decision delay (Table 4).

The results of the regression analysis are presented in Table 5, including standardized regression coefficients (βs) and the change in *R*^2^ for each predictor. The model accounted for 32% of the total variance in prehospital delay, *F*(9, 97) = 5.06, *p* < 0.001. Knowledge of cardiovascular risk factors, knowledge of ACS symptoms, and attributing symptoms to a cardiac cause accounted for 19.4, 11.5, and 10.1%, respectively of the variability, whereby more accurate knowledge of cardiovascular risk factors, correct attributions of symptoms to a cardiac cause, and more accurate knowledge of ACS symptoms were related to shorter decision delay. The other predictors were not significant (*ps* > 0.05).

**TABLE 5:** Linear regression analyses to determine the influence of each predictor on decision delay.

	**Decision delay (log)**				
	***B***	***SE***	**β**	***p***	***R*^2^**
Knowledge of cardiovascular risk factors	−0.060	0.018	−0.312	0.001	0.19
Knowledge of ACS symptoms	−0.048	0.017	−0.254	0.006	0.12
Attribution to a cardiac cause (0 = no; 1 = yes)	−0.280	0.139	−0.189	0.047	0.10
Perceived severity of symptoms	−0.024	0.038	−0.058	0.540	0.07
Age	0.001	0.007	0.012	0.888	0.01
Gender (0 = male; 1 = female)	0.022	0.140	0.014	0.876	0.02
Education	−0.035	0.047	−0.067	0.458	0.04
Type of ACS (0 = non-STEMI; 1 = STEMI)	−0.047	0.129	−0.033	0.716	0.00
Days elapsed between cardiac event and questionnaire	−0.020	0.028	−0.065	0.479	0.00

**FIGURE 2:**
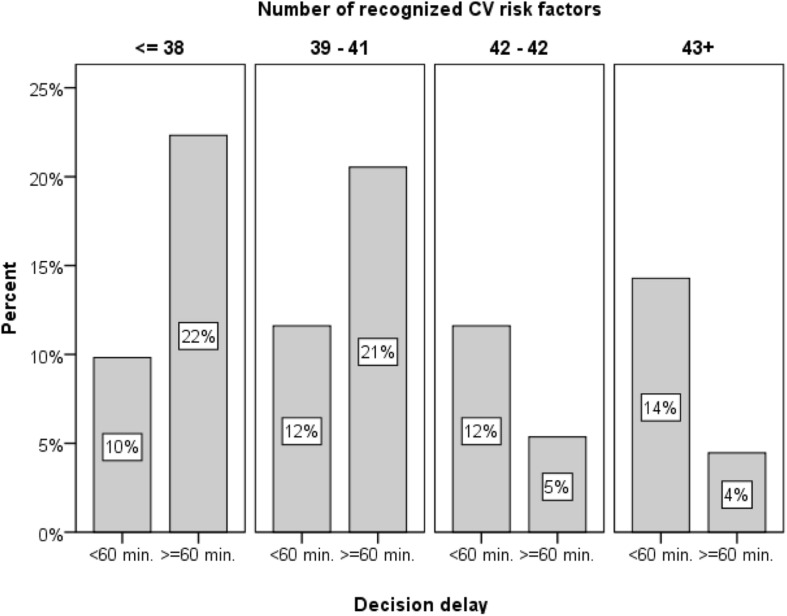
Percentage of patients waiting more than an hour to seek help after symptom onset as a function of cardiovascular symptom knowledge quartiles.

To illustrate the effects of the significant variables in the model, we considered the percentage of patients who waited more than 60 min to seek help after symptom onset, which is considered the “golden time window” for initiating treatment (Moser et al., 2006). Figure 2 displays this percentage as a function of cardiovascular risk factor knowledge quartiles, showing that the percentage of patients waiting more than 60 min is significantly higher for patients with relatively lower knowledge of risk factors (i.e., knowledge below the median). In the case of ACS symptom knowledge, the protective effect was observed in the highest quartile, in which only 28% waited more than 60 min, compared to an average of 60% in the lower quartiles. Finally, among those who attributed symptoms to a cardiac cause, only 30% waited more than 60 min, compared to 62% among those who did not attributed symptoms to a cardiac cause.

## Discussion

To the best of our knowledge, this is the first study showing that patients’ correct identification of cardiovascular risk and protective factors is related to shorter decision delays in seeking for help. This effect was independent of demographics and clinical characteristics and other important decision making factors such as symptom recognition and perceived severity of symptoms.

Previous research shows that people consistently underestimate the probability of experiencing negative outcomes (e.g., a disease). That is, they often show unrealistic optimism bias, which can reduce the accuracy of their risk appraisals and delay their help-seeking behavior (Weinstein, 1982; Blumenthal-Barby and Krieger, 2015). Previous research further showed that ACS patients tend to be overly optimistic regarding their risk of cardiovascular events (Dracup et al., 2008; Alfasfos et al., 2016; Thakkar et al., 2016). The current study raises the possibility that more accurate knowledge of cardiovascular risk factors might contribute to better decision making in patients with ACS by accurately increasing their perceived risk of suffering a cardiac event (Lefler and Bondy, 2004; Darawad et al., 2016), reducing the typical unrealistically optimistic perceptions of these patients (Dracup et al., 2008; Alfasfos et al., 2016; Thakkar et al., 2016).

Another potential mechanism behind the effect of risk factor knowledge could be related to the “representativeness heuristic” (Kahneman and Tversky, 1972; Blumenthal-Barby and Krieger, 2015). According to this heuristic, the probability of an event (e.g., a person experiencing a heart attack) is inferred by comparing it to an existing prototype (e.g., the typical person who would suffer a heart attack). If people have relatively high knowledge of cardiovascular risk factors, they could detect the similarity between their own characteristics and those of a prototypical person who develops cardiovascular disease. This could increase the perceived probability of suffering an important cardiac event and speed up help-seeking. For instance, a male smoker toward the end of his 60s, who knows that male gender, older age, and smoking are risk factors for developing cardiovascular diseases may be more likely to identify himself as someone who is likely to have a heart attack and thus make the decision to seek help sooner after symptom onset. This hypothesis should be investigated in future research.

Among the factors identified in previous research in relation to patient decision making during ACS is statistical numeracy: the ability to understand the mathematics of risk, including proportions, percentages, or probabilities (Cokely et al., 2014). More numerate patients were found to be three-to-four times more likely to have sought medical attention within 1 h after symptoms onset, independent of other cognitive, clinical, and demographic factors known to influence decision delay such as age and symptom severity (Petrova et al., 2017). Numeracy has been related to better knowledge and comprehension in diverse health contexts (Garcia-Retamero et al., 2019). It is possible that having more accurate knowledge of cardiovascular risk factors and more calibrated risk perceptions are among the mechanisms that make persons with higher numeracy more risk literate decision makers (Cokely et al., 2018). Future research can test this hypothesis in patients with ACS or other diseases.

In the current study, ACS patients from a hospital in Spain showed relatively good knowledge of cardiovascular risk factors. This result is in contrast with previous research in healthy and patient populations conducted in other countries showing that people tend to have very limited knowledge of their cardiovascular risk factors (Erhardt and Hobbs, 2002; Jensen and Moser, 2008; Wartak et al., 2011). Given these discrepancies, it would be best to further investigate the relationship between knowledge of risk factors and prehospital decision delay in other more diverse samples that also show more representative knowledge levels across the whole continuum of the scale. The risk factors questionnaire used in the current study was very detailed, including clinical, lifestyle, demographic, and psychosocial risk factors. The modifiable lifestyle and clinical factors were among the most recognized by participants – a result that it is not surprising given that they are the more frequently addressed in medical consultations and prevention efforts (ESC guidelines; Roffi et al., 2016). In contrast, many of the psychosocial factors are likely to be known only to medical professionals and researchers. Nevertheless, the current results show that many patients have correct intuitions regarding some of these factors, including the effect of stress, social isolation, and emotional tendencies (Table 3). These lay perceptions could be formed based on personal experience (e.g., based on the circumstances of family members or acquaintances who have suffered cardiovascular disease) or provided by physicians or the media. However, it is noteworthy that age, which is one of the strongest predictors of cardiovascular risk, was less recognized than many lifestyle factors – a result that suggest that interventions that effectively improve knowledge about cardiovascular risk facts might be useful.

In addition, results regarding symptom attributions are consistent with our previous findings (e.g., Arrebola-Moreno et al., 2020a), showing that patients who attributed their symptoms to a cardiac cause waited less to seek help. In contrast, the results regarding knowledge of ACS symptoms (i.e., that only very high knowledge appeared to have a protective relationship with decision delay) add evidence to a large number of previous studies showing mixed results (Petrova et al., 2017; Arrebola-Moreno et al., 2020a).

Such mixed findings could be due to the limitations of the retrospective methodology often used to study prehospital delays (see Arrebola-Moreno et al., 2020a, for more details). Patients recently diagnosed with ACS are often recruited shortly after the cardiac event to fill in a questionnaire. This methodology promotes several biases including memory biases (e.g., mild cognitive impairment is common after ACS, Saczynski et al., 2017), and selection biases (e.g., only survivors and clinically stable patients are included, thereby excluding the most vulnerable population). To illustrate, patients may not correctly remember exactly when their symptoms started or may not have interpreted the initial bodily sensations related to the cardiac episode as symptoms. Another limitation of this methodology is that it does not allow to control for the effect of learning (i.e., patients might learn from their experience with the disease). For instance, patient knowledge of symptoms could be influenced by the symptoms experienced during the cardiac episode and knowledge of risk factors could be influenced by their interaction with healthcare professionals, showing the hindsight bias, which is often referred to as the “I-knew-it-all-along” phenomenon (Christensen-Szalanski and Willham, 1991).

Despite these limitations, cross-sectional retrospective studies remain one of the most useful methods to study decision delay in ACS in a naturalistic setting. Unfortunately, prospective studies on prehospital delay, in which potential predictors of delay are recorded at baseline, are rare due to practical and financial issues stemming from the need to follow-up a very large number of individuals. In addition, factors directly related to decision making, such as perceptions and interpretations in the context of experiencing symptoms can only be recalled retrospectively (it could be impractical and even unethical to collect data during the experience of ACS).

As an alternative, studies with healthy populations in which participants report hypothetical decision delays could eliminate some of these biases and allow to study decision making processed in more detail (Arrebola-Moreno et al., 2020a). In this type of studies decision theories and knowledge regarding heuristics and biases could be used to understand and potentially improve patient decision making. Hence, we would like to encourage researchers from these fields to use their valuable expertise to solve this pressing societal problem. Coronary heart disease is the leading cause of death in Europe, causing about 1,739,000 deaths every year, which is 20% of all deaths (Wilkins et al., 2017).

Most interventions that aimed to improve patient decision making during ACS have focused on improving the recognition of symptoms in the population, raising awareness about successful treatment options, and giving instructions about what to do in case of symptoms (Mooney et al., 2012; Farquharson et al., 2019). Should the role of knowledge of cardiovascular risk factors be confirmed in future studies, then raising awareness about cardiovascular risk factors should be considered as a strategy in interventions and campaigns targeting patient delays during ACS.

## Data Availability Statement

This research is part of project “PySCA: Study on the impact of psychological factors in acute coronary syndrome” (Principal Investigator: JR-H). Individualized data from the project cannot be publically shared on a data repository due to the conditions of non-disclosure described in the hospital consent form signed by the participating patients. Study materials and detailed statistical results are available on the Open Science Framework (OSF) (10.17605/OSF.IO/CEHN7) and individualized data can be requested from the corresponding author.

## Ethics Statement

The studies involving human participants were reviewed and approved by the Ethics Committee of the University Hospital Virgen de las Nieves in Granada, Spain. The patients/participants provided their written informed consent to participate in this study.

## Author Contributions

DP, AC, JR-H, and RG-R conceived the study and prepared the study materials. DG and JR-H collected the data. DG managed the data and conducted the analyses together with DP. DG and DP wrote the first draft of the manuscript with input from all authors. All authors contributed to the interpretation of the results, provided critical revisions of the first draft, and approved the final version of the manuscript.

## Conflict of Interest

The authors declare that the research was conducted in the absence of any commercial or financial relationships that could be construed as a potential conflict of interest.

## Footnotes

1 The survey contained a second part addressing a different research question regarding willingness to adhere to lifestyle recommendations, which will be reported elsewhere.

2 gpower.hhu.de

3 The fictitious causes were used as filler items. These were factors that the accumulated scientific evidence had discarded as potential contributors to cardiovascular risk and risk factors for other diseases such as cancer or infectious diseases that have no important bearing on cardiovascular risk.
